# Cell intrinsic Wnt/β-catenin signaling activation

**DOI:** 10.18632/aging.101455

**Published:** 2018-05-22

**Authors:** Dominic B. Bernkopf, Jürgen Behrens

**Affiliations:** 1 Experimental Medicine II, Nikolaus-Fiebiger-Center, Friedrich-Alexander University Erlangen-Nuremberg, 91054 Erlangen, Germany

**Keywords:** PGAM5, mitochondria, Wnt

The Wnt/β-catenin signaling pathway is an evolutionary conserved mitogenic signaling pathway involved in embryonic patterning of body axes, regulation of stem cell fate and tissue homeostasis [1]. Deregulation of the pathway is associated with various diseases, e.g. colorectal carcinogenesis. The activity of the pathway is predominantly regulated via the cellular amounts of the transcriptional co-factor β-catenin. β-Catenin levels are tightly regulated by the β-catenin destruction complex consisting of the tumor suppressor adenomatous polyposis coli (APC), the scaffold protein axin and the two kinases casein kinase 1 α (CK1α) and glycogen synthase kinase 3 (GSK3) [1]. In this complex, β-catenin gets phosphorylated leading to its subsequent ubiquitination and proteasomal degradation. Binding of extracellular Wnt ligands to frizzled and LDL receptor related protein 5/6 (Lrp5/6) receptor pairs inhibits β-catenin degradation thereby activating transcription of β-catenin target genes [1]. Thus, the Wnt/β-catenin pathway, like other classical signaling pathways, is typically activated in a cell extrinsic fashion.

Recently, we identified a cell intrinsic way to activate the Wnt/β-catenin pathway via a signaling axis from mitochondria to β-catenin [2]: Loss of the mitochondrial membrane potential, which is critical to drive ATP synthesis, in damaged or stressed mitochondria triggers cleavage of the mitochondrial transmembrane phosphatase PGAM family member 5 (PGAM5) by the protease presenilin associated rhomboid like (PARL) [3]. We could show in biochemical and immunofluorescence-based experiments that cleaved PGAM5 is released into the cytosol from damaged mitochondria where it interacts with the β-catenin destruction complex-component axin [2]. Here, in the heart of the destruction complex, axin links the phosphatase PGAM5 to phospho-β-catenin thereby enhancing direct dephosphorylation of β-catenin by PGAM5, and counteracting CK1α- and GSK3-mediated β-catenin phosphorylation. These effects were significantly reduced in *PGAM5* and *PARL* knockout cells [2]. Together, we could show that PGAM5 released from damaged mitochondria activates Wnt/β-catenin signaling cell intrinsically via direct dephosphorylation of β-catenin.

That the Wnt/β-catenin pathway can be activated by both extrinsic and intrinsic mechanisms is reminiscent of the way cellular apoptosis is induced. Also apoptosis can be triggered extrinsically by the binding of FAS ligands to FAS cell surface death receptors, and cell intrinsically via the release of cytochrome c from mitochondria [4]. In both pathways, mitochondria are used for compartmentalization of key downstream effectors leading to separation of PGAM5 from β-catenin in case of Wnt signaling, and of cytochrome c from the apoptotic peptidase activating factor 1 (APAF1) in case of apoptosis [2,4]. While mechanisms of cytochrome c release via Bcl2/Bak family controlled outer membrane channels are well studied, nothing is known about how PGAM5 leaves mitochondria. Intact PGAM5 has been localized to both outer and inner mitochondrial membranes. At which of these locations PGAM5 gets cleaved by PARL and whether pores exist that allow cleaved PGAM5 to exit to the cytosol remain interesting questions for future research. Interestingly, in one study PGAM5 was shown to be released from mitochondria into the cytosol after treatment of cells with the pro-apoptotic drug staurosporine [5]. In this case PGAM5 might have used opened apoptotic channels for exit.

Activation of Wnt/β-catenin signaling has previously been shown to stimulate mitochondrial biogenesis [6]. Since cell extrinsic activation of the Wnt/β-catenin pathway primarily initiates cell division, concomitant stimulation of mitochondrial biogenesis might help to provide sufficient amounts of mitochondria for the daughter cells. Also activation of Wnt/β-catenin signaling through stable expression of a cytosolic PGAM5 version increased the mitochondrial mass [2]. Induction of mitochondrial biogenesis by PGAM5 released from dysfunctional mitochondria might thus represent a feedback loop which regulates mitochondrial homeostasis via intrinsic Wnt pathway activation. Interestingly, PGAM5 has also a role in the induction of mitophagy which leads to removal of damaged mitochondria by a specialized form of autophagy [7]. This suggests that PGAM5 has a dual role acting in both mitochondrial biogenesis and destruction. There are strong links between mitochondrial physiology and aging. It remains to be determined whether the connection between Wnt signaling activation and mitochondrial biogenesis described here is involved in cellular aging processes. For instance, Wnt signaling was shown to alter the production of reactive oxygen species (ROS) as a consequence of increased mitochondrial numbers leading to increased senescence [6].
